# Adding cetuximab to paclitaxel and carboplatin for first-line treatment of carcinoma of unknown primary (CUP): results of the Phase 2 AIO trial PACET-CUP

**DOI:** 10.1038/s41416-020-01141-8

**Published:** 2020-11-25

**Authors:** Gunnar Folprecht, Karolin Trautmann, Alexander Stein, Gerdt Huebner, Michael Stahl, Stefan Kasper, Albrecht Kretzschmar, Claus-Henning Köhne, Viktor Grünwald, Ralf-Dieter Hofheinz, Katharina Schütte, Harald Löffler, Carsten Bokemeyer, Alwin Krämer, Arbeitsgemeinschaft Internistische Onkologie (AIO) - CUP Group

**Affiliations:** 1 Technische Universität Dresden/University Hospital Carl Gustav Carus, Medical Department I, Dresden, Germany; 2 University Medical Center Hamburg-Eppendorf, Hamburg, Germany; 3 oho! ostholstein-onkologie, Oldenburg i.H., Germany; 4 Evangl. Kliniken Essen-Mitte, Department of Medical Oncology, Essen, Germany; 5 West German Cancer Centre, University Hospital Essen, Department of Medical Oncology, Essen, Germany; 6 MVZ Mitte, Leipzig, Germany; 7 University Clinic for Internal Medicine, Oncology und Hematology, Oldenburg, Germany; 8 Department of Hematology, Hemostaseology, Oncology & Stem Cell Transplantation, Hannover Medical School, Hannover, Germany; 9 West-German Cancer Centre Essen, University Hospital Essen, Essen, Germany; 10 University Medical Center Mannheim, Tagestherapiezentrum am ITM, Mannheim, Germany; 11 Marienhospital, Stuttgart, Germany; 12 Clinical Cooperation Unit Molecular Hematology/Oncology, German Cancer Research Center (DKFZ) and Department of Internal Medicine V, University of Heidelberg, Heidelberg, Germany

**Keywords:** Cancer therapy, Cancer

## Abstract

**Background:**

Patients with carcinoma of unknown primary (CUP) have a dismal prognosis, even when treated with multi-agent chemotherapy. We hypothesised that adding the epidermal growth-factor receptor (EGFR) inhibitor cetuximab to standard first-line chemotherapy with paclitaxel and carboplatin would improve PFS and RR in unfavourable CUP.

**Methods:**

This open-labelled, multicentre Phase 2 study included patients with unfavourable, untreated adeno- or undifferentiated CUP. Patients were randomised to receive either paclitaxel/carboplatin (group A) or paclitaxel/carboplatin plus cetuximab (group B) every 3 weeks for a maximum of 6 cycles followed by cetuximab maintenance in group B. The primary endpoint was PFS in the two groups. Secondary endpoints were RR, toxicity and overall survival (OS).

**Results:**

One-hundred-and-fifty patients were randomised (group A = 72, group B = 78). The median PFS and OS for all patients were 3.8 and 8.1 months (95% confidence interval (CI): 2.9–4.8 and 6.8–9.5). There was no significant difference in PFS (3.7 vs 4.6 months, HR 0.98) or OS (8.1 vs 7.4, HR 1.1) between the two treatment groups. Response rate tended to be better for chemotherapy plus cetuximab compared to chemotherapy alone (22% vs 15%). Adverse events grade ≥3 were comparable between the two groups, except for significantly increased skin toxicity in the cetuximab arm.

**Conclusions:**

Cetuximab plus paclitaxel/carboplatin did not improve PFS, OS and RR in metastatic CUP compared to paclitaxel/carboplatin alone. Addition of cetuximab resulted in additional skin toxicity.

**Clinical trial registration:**

The study was registered at clinicaltrials.gov as NCT00894569.

## Background

Carcinoma of unknown primary (CUP) is a challenging oncological problem defined as a metastatic cancer without a clinically evident primary tumour. It accounts for approximately 3% of all newly diagnosed advanced cancers with declining incidence during recent years.^1^ Patients with CUP have a dismal prognosis with a median overall survival of less than 1 year.^2^ The poor prognosis of CUP probably results from the aggressive nature of the disease^3^ and from a commonly encountered delay in specific diagnosis.^4^

During recent years, there has been substantial progress in more accurately defining the tissue of origin of CUP tissue biopsies using gene expression profiling and more advanced immunohistochemistry.^5,6^ However, most patients with CUP still receive empiric chemotherapy. A commonly accepted first-line regimen is a platinum/taxane combination that yields response rates (RR) of around 20–30% with a median overall survival (OS) of 9–10 months in selected patients.^7–9^ Several clinical trials have aimed to improve treatment results by combining standard chemotherapy with novel, molecular targeted therapies.^10–12^

Cetuximab is an anti-epidermal growth-factor receptor (EGFR) monoclonal antibody approved for the treatment of squamous cell head and neck and RAS wild-type colorectal cancer. In combination with standard first-line chemotherapy regimens, it significantly improves clinical outcome in the treatment of metastatic squamous cell carcinoma of the head and neck^13^ and metastatic colorectal cancer.^14,15^ Besides, inhibition of EGFR with the small-molecule erlotinib has been registered for the treatment of pancreatic cancer.^16^ Occult lung and pancreatic cancers are the most commonly identified primary tumours at autopsy in patients with CUP.^17^ Therefore, we hypothesised that the inhibition of EGFR by adding cetuximab to standard first-line chemotherapy would improve RR and progression-free survival (PFS) in patients with CUP.

Here we report the results of the PACET-CUP study, a randomised, multicentre Phase 2 study evaluating the efficacy and safety of adding cetuximab to paclitaxel/carboplatin for first-line treatment of adeno- and undifferentiated CUP.

## Methods

### Patient eligibility

Patients with adeno- or undifferentiated CUP were eligible. CUP histology had to be confirmed by the local pathologist. Tumours displaying any immunohistochemistry pattern indicative for a specific entity (i.e. Her-2 positive or hormone-receptor positive corresponding to breast cancer or CK7-negative/CK20-positive suggestive of colorectal cancer, carcinomas with neuroendocrine differentiation) were excluded. The study protocol intended central pathological review. However, central analysis could only be performed in 20 patients. For all other cases, tumour material was not sufficient for central review.

Eligible patients had to meet the following criteria: WHO performance status 0–1, measurable tumour lesion according to RECIST, age >/= 18 years, adequate liver and renal function (defined as bilirubin ≤1.5 × upper-normal level (UNL), ASAT and ALAT ≤ 2.5 × UNL or in the case of liver metastases ≤5 UNL, serum creatinine ≤1.5 UNL) and adequate bone marrow function (defined as neutrophil count ≥1.5 × 10^9^/L, platelet count ≥100 × 10^9^/L and haemoglobin >5 mmol/l). Specific patient subsets with clinically favourable CUP as described in current guidelines^18^ were excluded: females with axillary-node metastasis or peritoneal carcinomatosis, as well as younger males (<50 years) with retroperitoneal or mediastinal lymph nodes as the predominant tumour site. A standard diagnostic approach was applied to rule out a definite primary tumour site. This approach consisted of physical examination, tumour marker screening (CEA, CA 19-9 and AFP; for female patients: CA 15-3 and CA 125; for male patients: PSA and ß-HCG), an oesophagogastroduodenoscopy and abdominal–pelvic and thoracic, contrast-enhanced spiral CT, MRI or PET examination. Female patients received a gynaecologic examination and mammography. All patients provided written informed consent.

### Study design

This multicentre, open-labelled, randomised Phase 2 study (registered at clinicaltrials.gov as NCT00894569) was performed at 13 sites throughout Germany. The study protocol was approved by local ethics committees of the participating centres and the competent authorities. Eligible patients were centrally randomised (1:1) to receive paclitaxel/carboplatin (group A) or paclitaxel/carboplatin plus cetuximab (group B) and stratified by age, sex, presence of liver metastases and LDH level (normal vs above normal). Patients in group A received paclitaxel 175 mg/m^2^ plus carboplatin at an area under the concentration time curve (AUC) of 5. Chemotherapy was repeated every 21 days for a maximum of 6 cycles. Therapy in group B consisted of the same paclitaxel/carboplatin regimen preceded by cetuximab. The first cetuximab infusion contained 400 mg/m^2^ followed by weekly doses of 250 mg/m^2^. After 6 cycles of chemotherapy, patients in group B received maintenance therapy with weekly cetuximab. Completion of all six cycles of therapy was not a mandatory prerequisite to start maintenance. Chemotherapy and cetuximab dose modifications and treatment alterations were done according to the manufacturer’s recommendation. Therapy continued until progression, unacceptable toxicity or withdrawal of consent. During the study, tumour evaluation according to Response Evaluation Criteria in Solid Tumours (RECIST 1.0)^19^ using CT or MRI had to be performed by the local investigator/radiologist every 8 weeks.

### Study objectives

The primary endpoint of the study was PFS rate at week 32 (~8 months) after randomisation. The PFS rate was defined as the proportion of patients alive with stable disease (SD), partial response (PR) or complete response (CR) according to RECIST (version 1.0).^19^ Secondary endpoints included median PFS, overall RR (proportion of patients with a CR or PR as their best measured response) and toxicity in the two treatment groups (according to NCI-CTC version 3.0), as well as OS time defined as the time from randomisation to death in the intention-to-treat (ITT) population.

### Statistical plan

Based on the results of a previous study by the AIO CUP working group,^9^ we assumed a PFS rate at 32 weeks of 24% with standard treatment and 46% with the cetuximab-combination therapy. With a scheduled interim analysis after 50 patients, a one-sided alpha error of 0.05 and a power of 85%, 150 patients were planned to be randomised.

We used the Kaplan–Meier method to analyse PFS and OS and to estimate their medians. Survival rates between the two treatment arms were compared using the log-rank and chi-square test. Logistic regression and Cox regression analyses were performed to describe treatment effects in the different subgroups. We conducted multivariate analysis stratified by treatment arm to determine the influence of any baseline parameter on PFS and OS. Multivariate analysis was repeated using both treatment arms as variables.

## Results

### Study population

Between March 15, 2010 and March 20, 2017, one-hundred-and-fifty patients were randomised to paclitaxel/carboplatin (group A, *n* = 72) or paclitaxel/carboplatin/cetuximab (group B, *n* = 78). Patient characteristics are summarised in Table 1.

**Table 1:** Baseline characteristics according to treatment arm.

	Arm A (Chemotherapy)	Arm B (Chemotherapy + cetuximab)	All patients
	*N* = 72	*N* = 78	*N* = 150
Age (median)	59 y.	62.5 y.	61 y.
Age > 65 y.	26 (36%)	33 (42%)	59 (39%)
Gender: male	32 (44%)	34 (44%)	66 (44%)
WHO PS 0	24 (33%)	34 (45%)	58 (39%)
WHO PS 1	47 (65%)	42 (55%)	89 (60%)
WHO PS 2	1 (1%)	0 (0%)	1 (1%)
Leucocytes > 10	29 (40%)	25 (32%)	54 (36%)
LDH > ULN	46 (64%)	53 (68%)	99 (66%)
Liver met.	38 (53%)	34 (44%)	72 (48%)
Lung met.	16 (22%)	20 (29%)	36 (28%)
Lymph-node met.	37 (51%)	41 (53%)	78 (52%)
Bone met.	6 (8%)	7 (9%)	13 (10%)
No. of met. sites*			
1 site	16 (22%)	11 (14%)	27 (18%)
2 sites	25 (35%)	24 (31%)	49 (33%)
3 sites	17 (24%)	26 (33%)	43 (29%)
4 sites	8 (11%)	11 (14%)	19 (13%)
5 sites	4 (6%)	3 (4%)	7 (5%)
6 sites	2 (3%)	3 (4%)	5 (3%)

*One metastatic site corresponds to one involved organ system (i.e. liver, lung…).

The median age of the whole study population was 61 years with slightly more women included (56%). All but one patient had WHO performance status 0 or 1. The number of involved organ sites, the presence of liver, lung, bone or lymph-node metastases was well balanced between the two treatment groups. Similarly, the percentage of elevated LDH or leucocyte counts was equally distributed. There was no significant correlation between the number of metastatic sites and elevated LDH, increased leucocytes or WHO performance status.

### Treatment duration and toxicity

The whole study population received a median of three cycles of chemotherapy [quartile range 2.0–6.0]. Twenty-seven patients in arm A (37%) and 24 patients in arm B (31%) completed all six cycles. Thirty patients in arm B (38%) started with cetuximab-maintenance therapy. In 6 patients, carboplatin and paclitaxel had to be stopped due to toxicity, and maintenance was started before completing all 6 cycles of chemotherapy. All treatment-related adverse events >/= grade 3 for the 2 study groups are summarised in Table 2. The most common higher-grade adverse event in both treatment groups was leukopenia, which tended to occur more often in the cetuximab group. Skin toxicity was the only adverse event >/= grade 3 that was significantly increased in the experimental arm (0% in arm A vs 18% in arm B).

**Table 2:** Toxicity grade ≥ 3 according to treatment arm.

Adverse events Grade ≥ 3	Arm A (chemo)		Arm B (chemo + cetux)	
	*N* = 72		*N* = 78	
Leukopenia/neutropenia	14	(19%)	23	(29%)
Febrile neutropenia	0	(0%)	2	(3%)
Thrombopenia	2	(3%)	4	(5%)
Anaemia	4	(6%)	5	(6%)
Skin toxicity	0	(0%)	14	(18%)
Mucositis	2	(3%)	2	(3%)
Diarrhoea	5	(7%)	5	(6%)
Nausea/vomiting	2	(3%)	2	(3%)
Increased liver enzymes	2	(3%)	1	(1%)
Other gastrointestinal events	3	(4%)	6	(8%)
Fatigue/decreased performance status	4	(6%)	7	(9%)
Thromboembolic events	4	(6%)	4	(5%)
Syncope/falls	0	(0%)	2	(3%)
Arrhythmia	0	(0%)	2	(3%)
Other cardiovascular events	5	(7%)	6	(8%)
Polyneuropathy	3	(4%)	4	(5%)
Hypersensitivity	2	(3%)	2	(3%)
Pain	12	(17%)	13	(17%)
Other neurological events	2	(3%)	2	(3%)
Hypomagnesaemia	0	(0%)	4	(5%)
Renal events	2	(3%)	0	(0%)
Other lab events	3	(4%)	2	(3%)
Ascites/pleural effusion	3	(4%)	2	(3%)
Infections	3	(4%)	5	(6%)
Other events	4	(6%)	2	(3%)

### Treatment efficacy

**Fig. 1:**
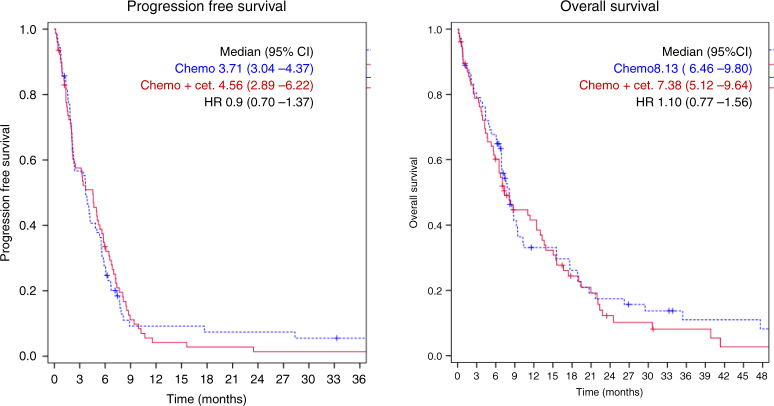
Progression-free and overall survival according to treatment arm. The graphs show the progression-free survival and overall survival probability for patients in arms A (blue dotted line) and B (red solid lines).

The PFS at week 32—which represented the primary endpoint—was 19.5% [95% CI: 10.5–28.5%] in patients receiving chemotherapy plus cetuximab (arm B) compared to 12.9% [95% CI: 4.5–21.3%] in patients receiving chemotherapy alone (arm A). This difference did not reach statistical significance. Similarly, the median PFS and OS were not significantly different between the two treatment arms (Table 3 and Fig. 1). The tumour-response rate in arm B was 22% compared to 15% in arm A, which was not significantly different.

**Table 3:** Treatment efficacy.

	Arm A (Chemo)	Arm B (Chemo + Cet)	All patients
	*N* = 72	*N* = 78	*N* = 150
*Tumour response*			
PR	**11**	**17**	**28**
	**15%**	**22%**	**19%**
95% CI:	7.9–26%	13–33%	13–26%
SD	25	25	50
PD	31	32	63
NA	5	4	9
*Progression-free survival*			
Median	**3.71**	**4.56**	**3.84**
95% CI	3.04–4.37	2.89–6.22	2.90–4.77
HR	**0.98**	95% CI: 0.70–1.37	
*Overall survival*			
Median	**8.13**	**7.38**	**8.13**
95% CI	6.46–9.80	5.12–9.64	6.81–9.46
HR	**1.10**	95% CI: 0.77–1.56	

Bold values indicate statistical significance.

### Subgroup analysis

**Fig. 2:**
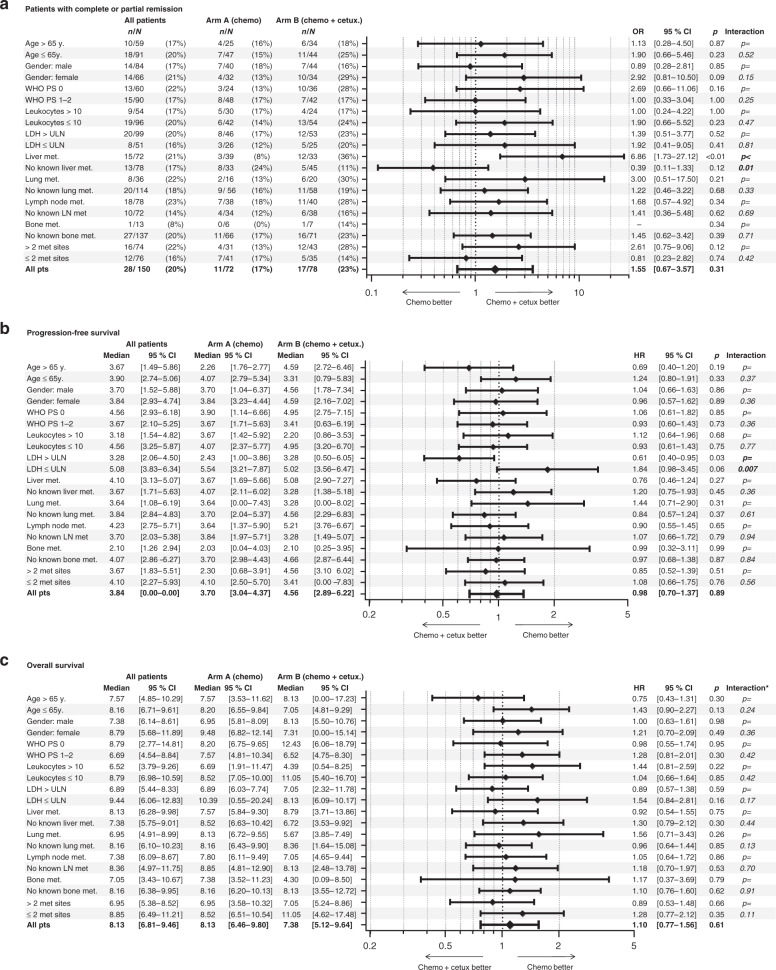
Treatment efficacy according to subgroups. The Forrest plots describe the Oddʼs ratio for patients with complete or partial remission (**a**) and the Hazard ratio for progression free survival (**b**) or overall survival (**c**) according to the baseline parameters (univariat analysis). * interaction between baseline parameter and treatment arm.

The subgroup analysis according to baseline characteristics demonstrated a higher response to chemotherapy plus cetuximab than to chemotherapy alone in patients with liver metastases (odds ratio 6.86 [95% CI: 1.73–27.12]) and a longer PFS with cetuximab in patients with elevated LDH (hazard ratio 0.61 [95% CI: 0.40–0.95]). There was no significant interaction between baseline parameters and treatment group with regard to overall survival for any subgroup analysed (Fig. 2).

A multivariate analysis stratified by treatment arm was performed to determine the influence of baseline characteristics on PFS, OS and RR. Elevated leucocyte count and impaired performance status were significantly associated with poorer OS. An elevated LDH correlated with a shorter PFS (Table 4). Adding the treatment arm as a variable did not change the results.

**Table 4:** Multivariate analysis for progression free survival and overall survival.

	Progression-free survival			Overall survival		
	HR	95% CI		HR	95% CI	
Lung met.	1.22	[0.80–1.87]	*p* = 0.35	1.38	[0.88–2.18]	*p* = 0.17
Lymph-node met.	0.99	[0.69–1.42]	*p* = 0.97	1.25	[0.86–1.81]	*p* = 0.25
Bone met.	1.62	[0.88–2.99]	*p* = 0.12	1.69	[0.92–3.08]	*p* = 0.09
Liver met.	0.93	[0.63–1.36]	*p* = 0.69	1.07	[0.71–1.61]	*p* = 0.74
Leucocytes > 10	1.20	[0.83–1.75]	*p* = 0.33	1.54	[1.04–2.26]	***p*** = **0.03**
LDH > ULN	1.62	[1.09–2.39]	***p*** = **0.02**	1.26	[0.86–1.85]	*p* = 0.23
Age > 65	1.10	[0.75–1.60]	*p* = 0.64	1.00	[0.68–1.47]	*p* = 0.99
WHO PS 1–2	1.22	[0.85–1.74]	*p* = 0.29	1.67	[1.13–2.46]	***p*** = **0.01**
Gender (male)	1.14	[0.78–1.66]	*p* = 0.49	1.19	[0.81–1.74]	*p* = 0.38
No of met sites > 2	1.14	[0.78–1.66]	*p* = 0.49	1.12	[0.76–1.66]	*p* = 0.58

Bold values indicate statistical significance *p* value <0.05.

The tables demonstrate the results of the multivariate analysis for the progression free survival and overall survival.

## Discussion

To our knowledge, this is the second largest clinical trial prospectively comparing the addition of a targeted agent to standard chemotherapy in patients with unfavourable CUP. There was no clinically relevant benefit from the combination of cetuximab and paclitaxel/carboplatin compared to paclitaxel/carboplatin alone despite numerically, but not statistically significant higher response and PFS rates at 8 months.

A smaller, non-randomised trial, including 60 patients with CUP, previously evaluated concurrent EGFR and vascular endothelial growth-factor (VEGF) blockage by adding both, erlotinib and bevacizumab to chemotherapy with paclitaxel/carboplatin.^12^ In agreement with our results, therapy was well tolerated with no new or unexpected side effects identified. The authors described an overall RR of 53% and a PFS of 38% at 1 year in their patient population. Another, more recent trial found that the addition of everolimus to carboplatin and paclitaxel resulted in a RR of 36% and a median PFS of 4.1 months.^10^ Of note, both studies were non-randomised. Another randomised Phase 2 trial showed that the addition of belinostat, a histone deacetylase inhibitor to paclitaxel/carboplatin, did not improve PFS of patients with CUP who were receiving first-line therapy.^11^

The median PFS of 3.8 months observed across treatment groups in our study is in agreement with other published data. Table 5 summarises available results from the most important clinical trials evaluating carboplatin/paclitaxel plus/minus experimental agents for the treatment of CUP. Since our trial included 150 patients from 13 different centres throughout Germany, we believe that our results are a good reflection of real-world data.

**Table 5:** Trials in patients with CUP treatment with carboplatin/paclitaxel combinations.

	Therapy	Phase	Response rate		PFS (months)	OS (months)	Statistical significance
			(*n*)	(%)			
Huebner et al.^9^	Carboplatin + paclitaxel vs gemcitabine + vinorelbin	2	10/42 9/45	23.8 20	6.1 (4.4–7–7) 3.2 (2.2–4.8)	11 (6.9–13.1) 7 (4.6–11.9)	Not tested (2-armed phase-2 trial)
Hainsworth et al.^12^	Carboplatin + paclitaxel + bevacizumab + erlotinib	2, single arm	35/60	53	8 (6.4–13.8)	12 (1–24)	-
Hainsworth et al. ^25^	Carboplatin + paclitaxel + etoposide vs gemcitabine + irinotecan	3	17/93 19/105	18 18	3.3 5.3	7.4 8.5	n.s.
Hainsworth et al.^11^	Carboplatin + paclitaxel vs carboplatin + paclitaxel + belinostat	2	9/43 19/42	21 45	5.3 (2.8–6.6) 5.4 (3.0–6.0)	9.1 (6.6–10.0) 12.4 (7.4–18.0)	n.s.
Hayashi et al.^22^	Carboplatin + paclitaxel vs site-specific therapy	2			4.8 5.1	12.5 9.8	n.s.
Yoon et al.^10^	Carboplatin + paclitaxel + everolimus	2, single arm	16/45	36	4.1 (2.8–5.7)	10.1 (7.3–14.8)	–

As in other types of cancer, specific mutations may increase sensitivity or result in resistance to anti-EGFR therapy. For example, in metastatic colorectal cancer, only tumours without mutations in KRAS and NRAS respond to therapy with anti-EGFR antibodies.^20^ It is possible that specific molecular CUP subsets might benefit from the addition of EGFR inhibition. However, due to insufficient tumour material in the majority of cases, we were not able to perform molecular analyses and collect this information. Techniques using circulating tumour DNA were not yet available when our study was conducted.

Since 2009, when the current study was designed, diagnostic approaches to classify CUP for clinical studies have changed substantially. Molecular tumour profiling helps to accurately predict the tissue of origin in many cases of CUP and might aid to select tumour site-specific therapies.^5,6,10,21^ However, in a randomised Phase 2 trial using gene expression profiling to enable site-specific treatment for patients with CUP, this approach did not result in a significant improvement of PFS or OS compared with empirical chemotherapy.^22^ Similarly, the results from a recently presented European Phase 3 trial including 243 patients did not show superior outcomes for patients with CUP treated with therapy tailored to the suspected primary site of origin as identified by molecular analysis.^23^ These studies are in line with a current meta-analysis that could not find a survival benefit for site-specific treatments in CUP.^24^

Adding cetuximab to standard chemotherapy failed to improve the clinical outcome of patients with CUP. With a median OS of 8.1 months across all patients enrolled, our trial confirms the poor prognosis of this disease and underlines the medical need for better treatment options. Clinical trials enabling early biomarker-driven targeted therapies by using more advanced molecular and immune-profiling techniques are underway.

## Data Availability

Access to the anonymised dataset can be requested from the corresponding author.
